# Chitosan from sea urchin (*Diadema setosum*) spines for orthodontic miniscrews: Antibacterial effects against key oral pathogens

**DOI:** 10.1016/j.jobcr.2025.02.014

**Published:** 2025-03-11

**Authors:** Karima Qurnia Mansjur, Eka Erwansyah, Ardiansyah S Pawinru, Mansjur Nasir, Arni Irawaty Djais, Virgino Calvine Sumule, Nurnabilla Syfadewi Attaya, Dian Yosi Arinawaty, Islamy Rahma Hutami, Andi Dian Permana

**Affiliations:** a Department of Orthodontics, Faculty of Dentistry, Hasanuddin University, Indonesia; b Department of Periodontology, Faculty of Dentistry, Hasanuddin University, Indonesia; c Department of Oral Biology, Faculty of Dentistry, Universitas Muhammadiyah Yogyakarta, Indonesia; d Department of Orthodontics, Faculty of Dentistry, Islamic University of Sultan Agung, Indonesia; e Faculty of Pharmacy, Hasanuddin University, Indonesia

**Keywords:** Chitosan, Sea urchin, Miniscrew

## Abstract

**Objective:**

Peri-implantitis, exacerbated microbial growth characterized by progressive bone loss and soft-tissue inflammation, significantly contributes to miniscrew failure during orthodontic treatment. Using a natural antibacterial coating presents an innovative approach to combat bacterial colonization. Sea urchin (*Diadema setosum*) spines containing chitosan (CS) exhibit notable antibacterial properties and biocompatibility effects. This study investigates the antimicrobial potential of CS from sea urchin spines applied onto the surfaces of orthodontic miniscrews, aiming to mitigate the impact of peri-implantitis.

**Materials and methods:**

The surface functional groups, phase composition, and crystal structure of CS were investigated using traditional examination methods alongside energy-dispersive X-ray analysis. The antibacterial activity of CS was evaluated against three bacteria by the disk diffusion method, minimum bacterial concentration (MBC), and minimum inhibitory concentration (MIC). Stainless steel miniscrews were coated with CS, and the surface was characterized by scanning electron microscopy (SEM).

**Results and discussion:**

Sea urchin-derived chitosan demonstrated significant antibacterial effects against key oral pathogens associated with peri-implantitis, with minimum inhibitory concentrations (MICs) of 16 ppm against *Fusobacterium nucleatum* and 32 ppm for both *Aggregatibacter actinomycetemcomitans* and *Porphyromonas gingivalis*. The minimum bactericidal concentrations (MBCs) were 4 ppm for *A. actinomycetemcomitans* and 16 ppm for both *F. nucleatum* and *P. gingivalis,* indicating its strong bactericidal potential. Scanning electron microscopy (SEM) revealed that sea urchin chitosan effectively adhered to the surface of orthodontic miniscrews, showcasing its potential as a functional antimicrobial coating. These results emphasize the capability of sea urchin chitosan to target key oral pathogens, offering a promising approach to enhance microbial resistance and improve outcomes in orthodontic treatments.

## Graphical abstract

**Figure:**
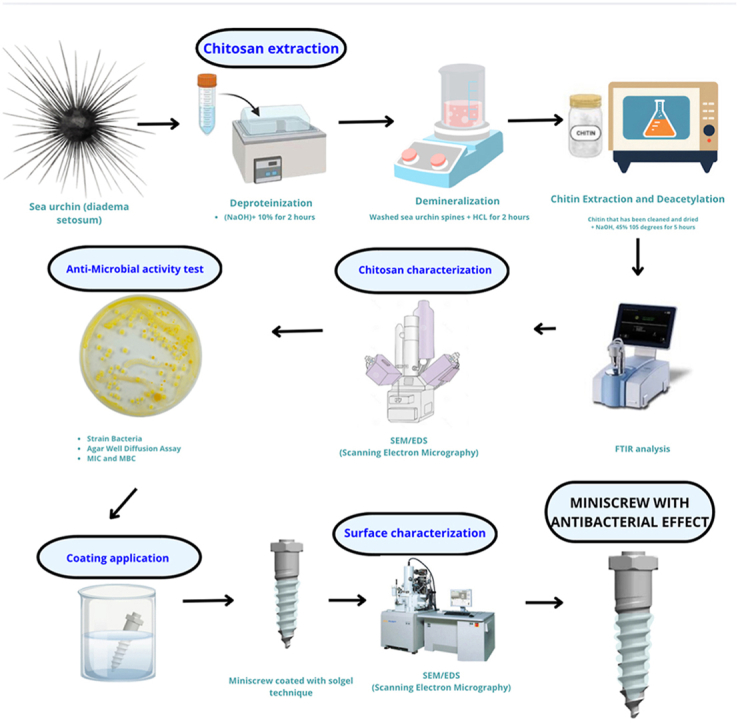

## 1 Introduction

Orthodontic miniscrews have emerged as crucial tools in modern orthodontic practices, providing temporary anchorage for various dental movements.^1^ These devices offer reliable support without patient cooperation, enabling secure anchorage for diverse tooth movements and facilitating directional changes previously unattainable with traditional orthodontic techniques.^2^ In contrast, the clinical utilization of miniscrews is associated with certain risks and concerns. These include potential issues such as miniscrew fractures, damage to peri-implant tissues, and overall miniscrew failure. Such complications may arise during the insertion of the screw, application of orthodontic forces, or removal of the screw, ultimately resulting in delays in treatment timelines.^3^^,^^4^ Alongside mechanical factors, biological considerations significantly influence the likelihood of failures. A particularly pertinent issue is peri-implantitis—a condition characterized by inflammation caused by infection around the miniscrew site—which stands out as a critical biological contributor to miniscrew failure.^5^^,^^6^ However, miniscrew failure is not solely driven by peri-implantitis. Other contributing factors include mechanical issues such as inadequate primary stability during insertion, improper placement technique, or excessive orthodontic loading, which may lead to micro-movements and miniscrew loosening. Soft tissue complications, such as gingival overgrowth, can further impede oral hygiene, promoting inflammation. Additionally, systemic conditions like diabetes, osteoporosis, and smoking can negatively impact osseointegration and tissue healing. Addressing these factors alongside bacterial resistance is crucial for ensuring the long-term success of orthodontic miniscrews.^7^, ^8^, ^9^

Pathogenic bacteria, such as *Fusobacterium nucleatum* (FN)*, Aggregatibacter actinomycetemcomitans* (AA), and *Porphyromonas gingivalis* (PG), primarily drive peri-implantitis.^10^^,^^11^ These bacteria form biofilms on the miniscrew surfaces, creating a complex, multilayered matrix that protects them from external threats. Bacteria can adapt better to unfavorable conditions within biofilms, making infections more persistent and challenging to treat. The biofilm matrix hinders the penetration of antimicrobial agents, allowing bacteria to survive and thrive even in hostile environments.^12^^,^^13^ Practical strategies to combat these bacterial infections are crucial for preventing peri-implantitis and ensuring the long-term success of orthodontic miniscrews.^14^

Combating bacterial biofilms is crucial for preventing peri-implantitis and reducing antibiotic reliance. A single dose of prophylactic antibiotics in healthy individuals can significantly select for resistant bacterial strains.^15^ Overuse of antibiotics fosters the development of resistant bacteria within the resident oral and gastrointestinal microflora, disrupting these communities and causing substantial disturbances in oral niches, posing a significant public health threat.^16^ Therefore, enhancing the miniscrews' surface properties to resist bacterial colonization and promote healthy tissue integration is a vital strategy.^9^^,^^17^

Bacterial resistance to conventional antibiotics poses a significant challenge in oral healthcare.^15^^,^^18^ In response to this issue, biopolymers such as chitosan have gained attention due to their inherent antibacterial properties, offering a promising alternative for preventing infection associated with orthodontics miniscrew.^19^, ^20^, ^21^ Chitosan, a biopolymer derived from chitin, is known for its excellent biocompatibility, biodegradability, and antimicrobial properties, making it highly valuable for biomedical applications.^22^^,^^23^ Among various sources, the sea urchin (*Diadema setosum*), which is abundant in Indonesian waters, stands out due to its high-quality chitin content, as well as calcium carbonate that enhances its utility in various applications.^24^ The chitin from these spines is noted for its purity, high molecular weight, antibacterial properties, and mechanical strength.^25^^,^^26^

This study evaluates chitosan coatings from *Diadema setosum* spines on orthodontic miniscrews to enhance biocompatibility and inhibit microbial growth. By leveraging this natural resource, the study aims to improve miniscrew functionality, combat peri-implantitis biofilms, and reduce antibiotic reliance. The findings could advance miniscrew coating, optimize orthodontic outcomes, and promote sustainable use of Indonesia's marine resources.

## 2 Material and methods

### 2.1 Ethical approval

This experiment was performed by the International Guidelines for the Care and Use of Laboratory Animals and plants. Clearance for this study was obtained from Hasanuddin University Dental Hospital, Makassar, Indonesia; Research Ethics Committee number: **018/KEPK-FKG-RSGMP-UH/EA/X2024.**

### 2.2 Preparation of chitosan from sea urchin (Diadema setosum) spine extract

Sea urchin spines (*Diadema setosum*) were collected from the waters surrounding the Makassar region of South Sulawesi, Indonesia. The extraction and preparation of chitosan from these sea urchin spines encompasses three primary stages: deproteination, demineralization, and deacetylation. In the initial phase of deproteination, the objective is to remove proteins embedded in the sea urchin spines. This involves weighing 100 g of sea urchin spines and combining them with a 3 N NaOH solution at a ratio of 1:10. The mixture is then heated to a temperature of 90 °C for 1 h while being stirred continuously. Following this heating process, the solution is filtered and thoroughly rinsed with distilled water until it reaches a neutral pH. The subsequent stage of the process, demineralization, is aimed at eliminating minerals from the previously processed material. The deproteinized product is treated with a 1 N HCl solution in a ratio of 1:7 and subjected to heating conditions that maintain a temperature of 90 °C for 1 h while ensuring continuous stirring. Following this treatment, thorough filtration and careful washing with distilled water were conducted to preserve neutrality in pH.

As a result of these processes, chitin remains predominant after drying. The final stage involves deacetylation; during this phase, the extracted chitin is immersed in a 50 % NaOH solution at an approximate ratio of 1:20 and heated to 140 °C for 2 h under continuous stirring. Subsequent treatments include extensive washing with distilled water to restore neutrality before undergoing another drying cycle. This meticulous series of procedures—deproteination, demineralization, and deacetylation—effectively yields high-quality extractable chitosan from sea urchin spines.^24^

### 2.3 Preparation of sea urchin spine extract at various concentrations

The preparation of sea urchin spine extract at specified concentrations of 0.4 %, 0.6 %, and 0.8 % is initiated by accurately weighing the necessary quantities: specifically, 0.4 g, 0.6 g, and 0.8 g for each respective concentration. Each measured quantity of the extract is subsequently diluted in a sterile petri dish with a total volume of 10 mL of saline solution. Following this dilution process, comprehensive stirring is performed to ensure uniformity across all samples, thereby establishing the desired variations in concentration.

### 2.4 Characterization of chitosan

#### 2.4.1 Fourier transform infrared spectroscopy (FTIR)

The transmission method was employed to evaluate the CS by grinding a 2 mg sample together with 100 mg of dried potassium bromide (KBr) and then compressing this blend into a disk that has a diameter of 3 mm. All analyses were conducted utilizing an IRPrestige-21 spectrometer from Shimadzu, with absorbance measurements recorded over the wavelength range of 400–4000 cm^−1^.

#### 2.4.2 Energi dispersive X-ray spectroscopy (EDX)

An energy-dispersive X-ray spectroscopy (DX-700HS Shimadzu, Japan) instrument was employed to determine the elemental composition of the samples. This apparatus is integrated with a scanning electron microscope (SEM) to gather elemental data of the specimens under investigation.

#### 2.4.3 Scanning electron microscopy (SEM)

The surface morphology of chitosan and miniscrew surfaces was examined utilizing a field scanning electron microscope (FSEM, JEOL, JCM-6000plus, Peabody, MA, USA). Before observation with the scanning electron microscope (SEM), the samples were subjected to gold sputter coating and analyzed at an accelerating voltage of 5 kV.

### 2.5 Antimicrobial activity of chitosan

#### 2.5.1 Bacterial preparation of Fusobacterium nucleatum, Aggregatibacter actinomycetemcomitans, and Porphyromonas gingivalis

Cultures of *Aggregatibacter actinomycetemcomitans, Fusobacterium nucleatum,* and *Porphyromonas gingivalis* were obtained from the Microbiology Laboratory at the Faculty of Pharmacy, Muslim Indonesia University. These cultures were inoculated in an anaerobic chamber maintained at 37 °C with a gas composition of 85 % nitrogen (N2), 10 % hydrogen (H2), and 5 % carbon dioxide (CO2) for 48 h. The culture was subsequently kept at room temperature for five days, continuously agitated with a magnetic stirrer.

#### 2.5.2 Well diffusion technique for antimicrobial assessment

In this study, the inhibitory effects of chitosan extract derived from sea urchin spines were evaluated in vitro against three bacterial strains. The experiment utilized various concentrations of chitosan (0.4 %, 0.6 %, and 0.8 %) along with a control group consisting of 0.1 % acetic acid as a negative control and azithromycin as a positive control. To determine the impact of chitosan on bacterial growth, we employed the well-diffusion method. Initially, a Petri dish was prepared with an initial layer comprising 10 mL of Nutrient Agar (NA) media. Following solidification, an additional layer containing 10 mL of NA media inoculated with the test bacteria was added, after which the setup was maintained under laminar airflow for 2 h to facilitate hardening and ensure proper bacterial diffusion. Subsequently, wells measuring 5 mm in diameter were created by removing portions from the agar medium. A total volume of 200 μL containing either chitosan or one of the control substances was introduced into each well. The Petri dishes were incubated at a temperature of 37 °C for three days (3 × 24 h), during which observations were conducted every 24 h to assess any inhibition zones or clear zones that emerged around each well. Measurements to quantify these inhibition zones were performed using calipers (mm), noting precise diameters around each well accordingly.^16^^,^^27^ Sterility controls, including blank wells without bacterial inoculation, were included in the antimicrobial assays to ensure the validity of the results.

#### 2.5.2 Minimum inhibitory concentration (MIC) and minimum bacterial concentration (MBC)

The MIC and MBCs of chitosan were evaluated using the microdilution method. A 100 μL aliquot of a 0.5 McFarland bacterial suspension, which corresponds to approximately 10^8 cells/mL, was inoculated into each well of a microtiter plate. Subsequently, tenfold serial dilutions of the were prepared at concentrations of 0, 0.25, 0.5, 1, 2, 4, 8, 16, 32, 64,128 and 256 μg/mL and then incubated for 24 h at a temperature of 37 °C.^28^

### 2.6 Chitosan coating Method for miniscrews Utilizing the Bumgardner Approach with adjustments

With some modifications, a chitosan layer was prepared via a chemically driven salinization reaction adapted from the Bumgardner protocol.^29^ The modifications included adjusting the reaction time and pH levels to optimize chitosan yield. The miniscrew was placed in a stirred solution comprising 5 % water and 95 % ethanol by volume, which had been acidified to a pH of 4.5 using 10 M acetic acid. Following this, a salt coupling agent at a concentration of 2 vol% was introduced for 10 min at room temperature. During this period, the pH was carefully maintained between 4.5 and 5.5 through adjustments made with either 1 M NaOH or 10 M acetic acid. Subsequently, the miniscrew underwent decontamination with ethanol before being preserved at an elevated temperature of 110 °C for 10 min. It was then suspended in a stirred solution containing glutaraldehyde at a concentration of 2 % volume and maintained at pH 4.3 overnight under ambient conditions. Concurrently, a chitosan solution with a concentration of 2 % weight was prepared by dissolving chitosan in a mixture containing 0.2 % acetic acid at room temperature; this solution was stored refrigerated at approximately 4 °C overnight.

An aliquot (1 ml) of the chitosan solution was subsequently applied over the implanted disc under ambient conditions and allowed to dry through evaporation over five to seven days to form a thin coating layer. The coated miniscrews were then sterilized by exposure to ultraviolet (UV) light for 1 h, followed by immersion in approximately saline aqueous ethanol (70 %) for 2 h and rinsed twice with phosphate buffer solution (PBS).

### 2.7 Data analysis

Data analysis was carried out using statistical tests. A normality and homogeneity of variance test were first carried out for the power obtained from the research results. This analysis employs the Shapiro-Wilk and Levene's tests to assess homogeneity of variances. The data followed a normal distribution with homogeneous variance, allowing for the execution of a One-Way ANOVA parametric examination. Moreover, the Tukey test was conducted to identify significant differences in the inhibitory effectiveness of sea urchin chitosan extract at different concentrations on the growth of *Fusobacterium nucleatum, Aggregatibacter actinomycetemcomitans,* and *Porphyromonas gingivalis bacteria.*

## 3 Results

### 3.1 Sea urchin chitosan FTIR test results

**Fig. 1:**
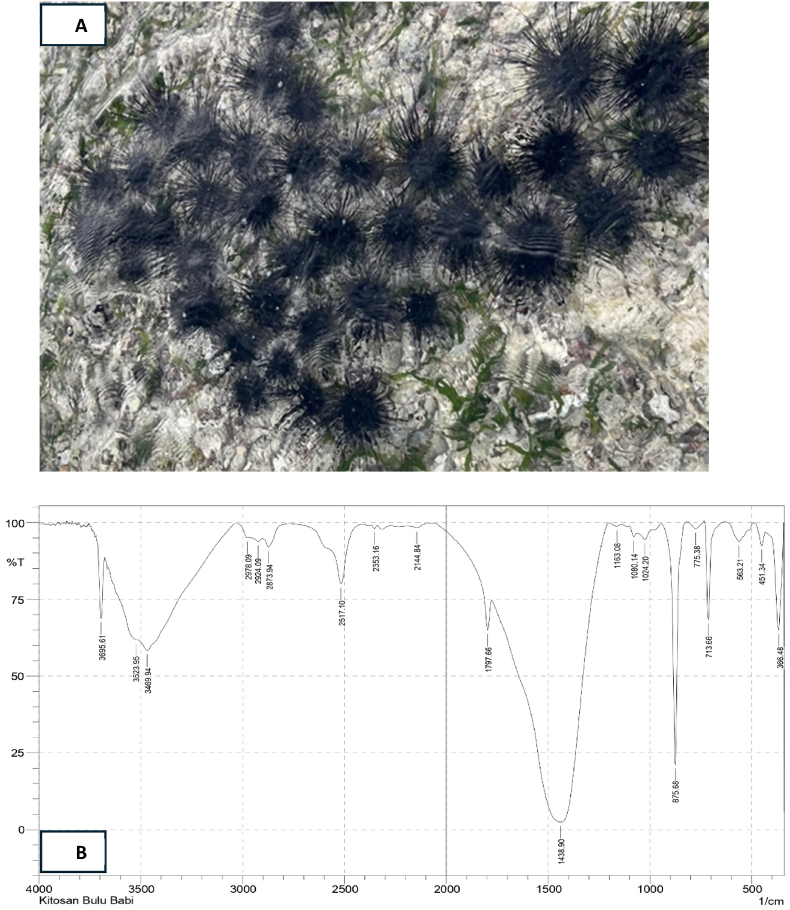
(A) Sea urchin spines (*Diadema setosum*) were collected from the waters surrounding the Makassar region of South Sulawesi, Indonesia. (B) Analysis of chitosan utilizing FTIR encompassing various components. These results confirm the presence of chitosan characteristics in the compound extracted from sea urchins.

The FTIR (Fourier Transform Infrared) analysis of chitosan extracted from sea urchins reveals several characteristic absorption bands (Fig. 1). An absorption band at 3402.68 cm^-1 indicates the presence of the N-H group. The band at 1421.64 cm^-1 suggests the presence of the CH3 group. The C-N group appears at wave numbers 1331.39 cm^-1 and 1024.22 cm^-1, while the C-O group is at 1153.91 cm^-1. Additionally, an absorption band at 2877.2 cm^-1 indicates aliphatic C-H stretching vibrations. Further FTIR analysis shows an absorption band at 3695.61 cm^-1, indicating the presence of the O-H group. The N-H group appears at 3469.94 cm^-1, and the aliphatic C-H group is detected at 2924.09 cm^-1. The CH3 group is identified at 1438.9 cm^-1, the C-N group at 1024.2 cm^-1, and the C-O group at 1080.14 cm^1. These results confirm the presence of chitosan characteristics in the compound extracted from sea urchins.

**Fig. 2:**
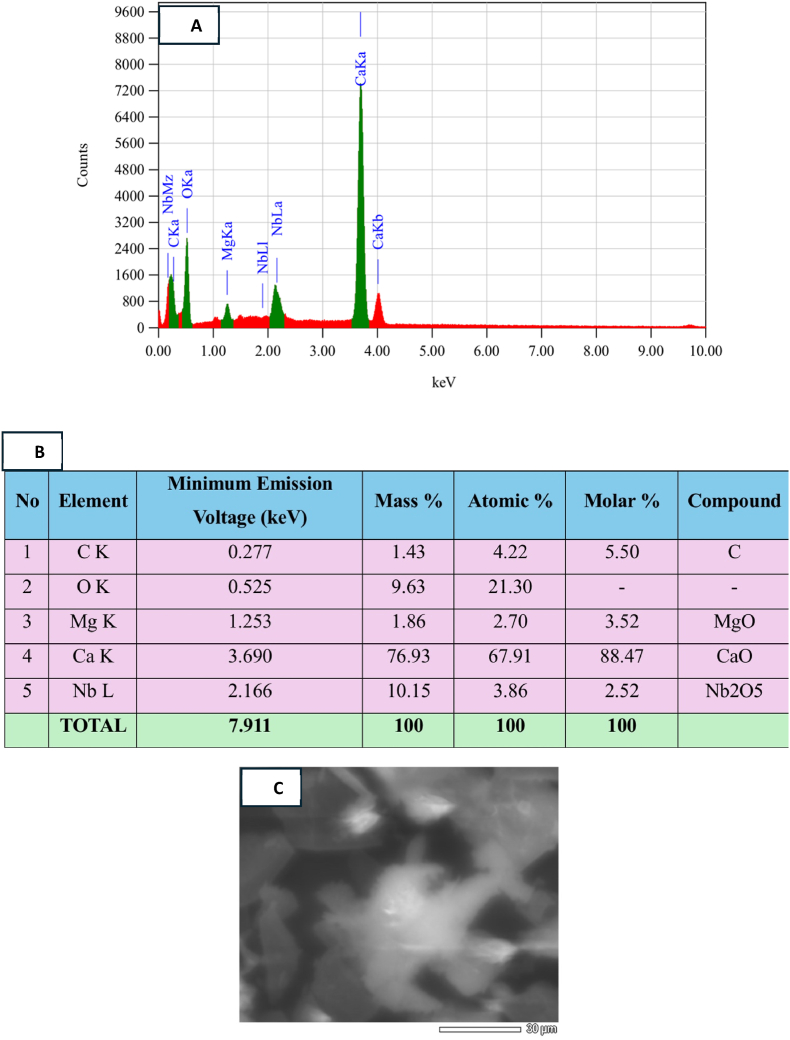
EDX analysis of chitosan powder. Fig. 2 (A) Illustrates the quantitative elemental composition and EDX spectra. (B) Analytical results indicated the presence of carbon (C), oxygen (O), magnesium (Mg), and calcium (Ca) in the chitosan samples. (C) The sea urchin spines exhibit a porous structure characterized by a non-homogeneous and irregular surface.

**Fig. 3:**
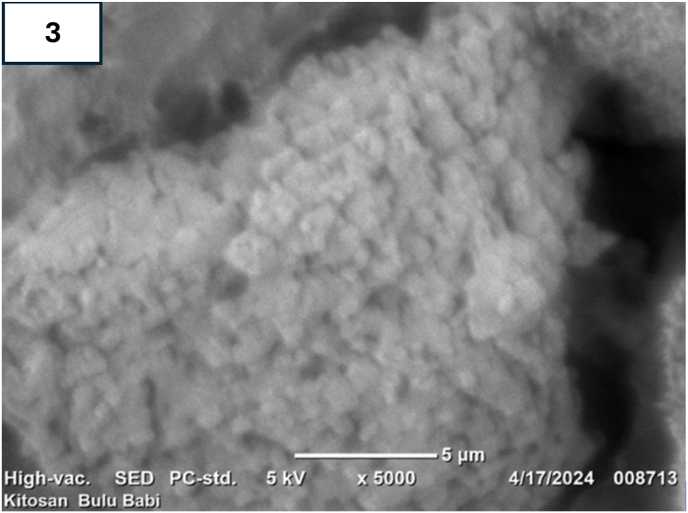
SEM of chitosan powder. Fig. 3 the morphology of chitosan powder was examined utilizing SEM with voltage set at 20 kV. The resulting micrographs demonstrated a uniform crystalline surface characterized by a dense structure, minimal voids, and a multitude of dimples, which suggest the presence of pores within the material.

### 3.2 Antibacterial activity of chitosan

**Fig. 4:**
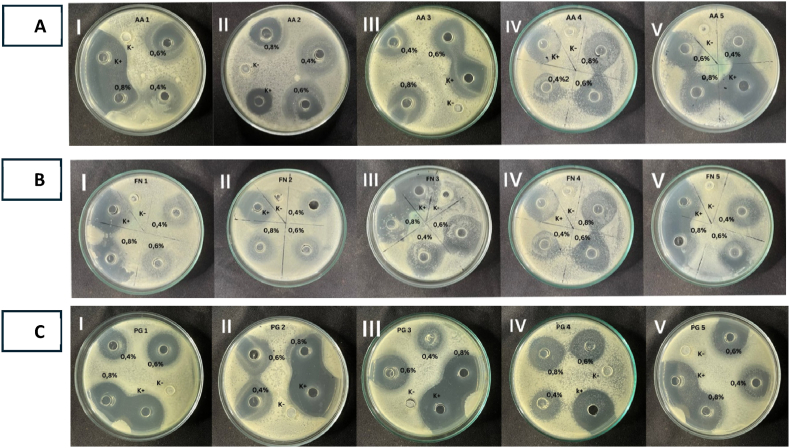
Antibacterial activity test results against *A. actinomycetemcomitans, F. nucleatum,* and *P. gingivalis*. (A, B, C) Each panel displays five repetitions (I, II, III, IV, V) of the antibacterial assay to assess the consistency and reliability of chitosan's antibacterial activity.

The chitosan from sea urchin spines showed good antibacterial activity against *A. Actinomycetemcomitans, F. nucleatum,* and *P. gingivalis* (Fig. 4A, B, C). The antibacterial activity was found to be concentration-dependent. At the same time, the activity of bacteria was absent in negative control.

The figures illustrate the antibacterial activity of chitosan derived from sea urchins against the bacteria *A. actinomycetemcomitans, F. nucleatum,* and *P. gingivalis*. The variations in the mean diameter of the inhibition zones indicate differences in antibacterial efficacy among the treatment groups. Notably, the group utilizing 0.1 % acetic acid as a solvent exhibited no antibacterial effect. In contrast, the azithromycin-treated group displayed significant inhibition zones measuring 20.2 ± 1.92 mm for *F. nucleatum*, 24.8 ± 5.35 mm for *P. gingivalis*, and a notably higher average of 33.4 ± 0.89 mm for *A. actinomycetemcomitans*, demonstrating its potent antibacterial properties as a comparative control.

**Fig. 5:**
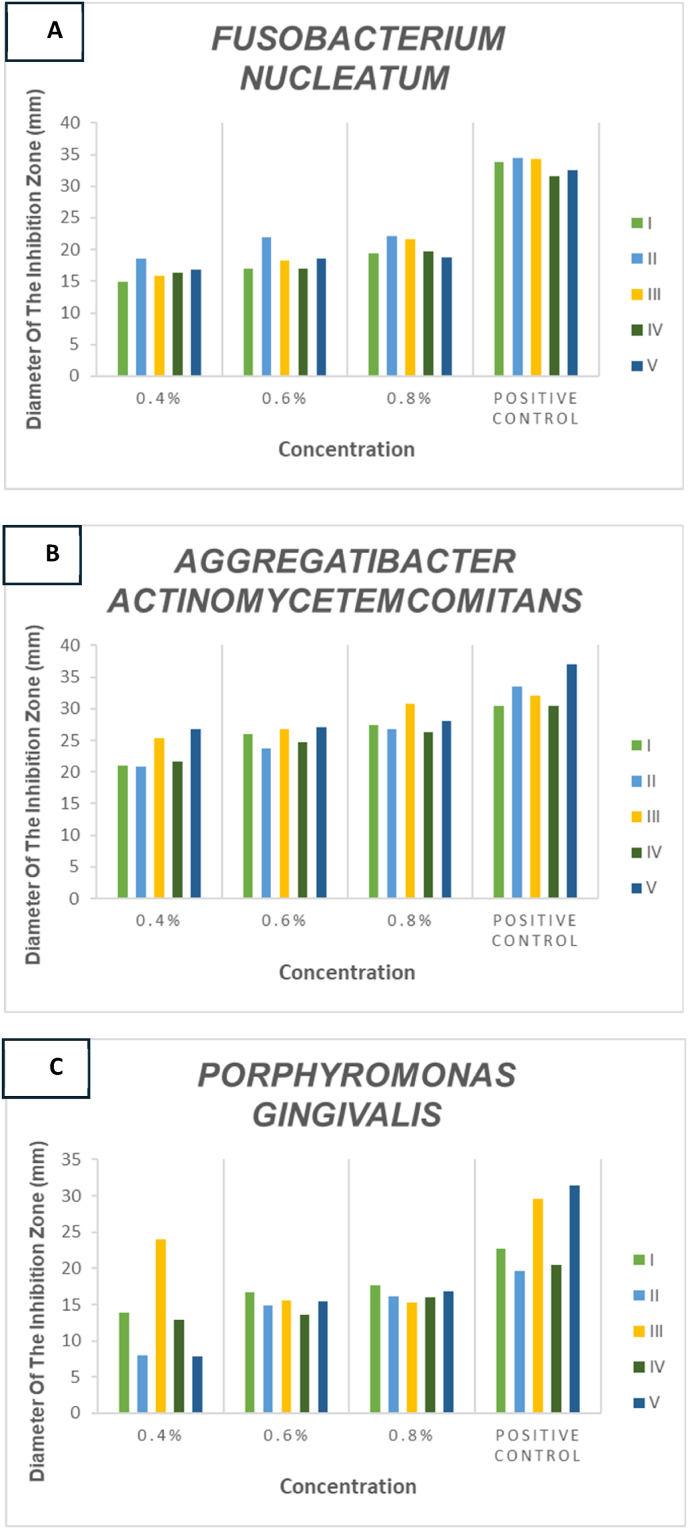
Average zone of inhibition of chitosan extract from sea urchin spines against *F. nucleatum* (5A), *A. actinomycetemcomitans* (5B), and *P. gingivalis* (5C).

Furthermore, chitosan extracts from sea urchins at concentrations of 0.4 %, 0.6 %, and 0.8 % resulted in observable inhibition zones against all three bacterial strains tested, confirming their antibacterial potential (Fig. 5A, B, C). The formation of the inhibition zone began at a concentration of 0.4 %, with mean values recorded as follows: 13.4 ± 6.54 for *P. gingivalis*, 14.40 ± 1.14 for *A. actinomycetemcomitans,* and a notably higher mean of 16.6 ± 1.51 for *F. nucleatum*. At a concentration of 0.8 %, the largest zones of inhibition were observed (excluding the positive control), yielding mean values of 19.2 ± 1.3 for *F. nucleatum*, 16.4 ± 1.14 for P. gingivalis, and an impressive mean value of 20.4 ± 1.51 for A*. actinomycetemcomitans*. The results presented in the table indicate that the size of the clear zone increases with higher concentrations applied, demonstrating that chitosan at a concentration of 0.8 % exhibits substantially more potent antibacterial activity compared to both the lower concentrations of 0.4 % and 0.6 % (Table 1). A graphical representation is provided below to illustrate these comparative concentrations further.

**Table 1:** Inhibition zone mean (mm) at different concentrations.

Concentration	Inhibition Zones Mean (mm) Chitosan Sea Urchins		
	*Fusobacterium nucleatum* (FN)	*Aggregatibacter actinomycetemcomitans* (AA)	*Porphyromonas gingivalis (PG)*
0,4 %	16.6±1.51^c^	14.4±1.14^c^	13.4±6.54^c^
0,6 %	18.6±2.07^c^	17±4.84^c^	15.4±1.14^c^
0,8 %	20.4±1.51^b^	19.2±1.3^c^	16.4±1.14^c^
Azitromychin (positive control)	33.4±0.89^b^	20.2±1.92^b^	24.8±5.35^b^
Acetic acid 0,1 % (negative control)	0		

^a^Each data point represents ± standard deviation.

^b^David and Stout Category; Strong.

^c^David and Stout Category; Moderate.

Based on the inhibitory strength category according to David & Stout,^30^ the inhibitory strength of chitosan compounds from sea urchin spines with concentrations of 0.4 % and 0.6 % *against A. actinomycetemcomitans* and *P. Gingivalis* is categorized as moderate, and in *F. Nucleatum,* the concentration of 0.8 % is categorized as strong.

According to the data presented in the table above, significant differences have been observed between the 0.4 % concentration group and the 0.8 % concentration group for both *F. nucleatum* and *A. actinomycetemcomitans*. In contrast, no significant differences were identified within the *P. gingivalis* group, except comparisons made against control groups. There were no significant differences found between 0.4 % and 0.6 %, as well as 0.6 % and 0.8 % (see Table 2).

Based on the MIC test results for chitosan extract from sea urchin spines against *F. nucleatum* (Table 3), the MIC was determined to be 16 ppm, indicated by a color change to red after the addition of TTC and incubation for 1 h. The lowest concentration showing a clear, non-pink solution was identified as the MIC. The color change occurs due to the reduction of the TTC reagent by the dehydrogenase enzyme produced by living bacteria, forming a red/pink triphenylformazan compound. Following the MIC determination, the chitosan extract was cultured on Nutrient Agar to assess the MBC. The MBC is defined as the lowest concentration of the extract that kills 99.9 % of the bacterial growth. The MBC results varied among the three bacteria, with values of 4 ppm for *A. actinomycetemcomitans*, 16 ppm for *F. nucleatum*, and 64 ppm for *P. gingivalis* (Table 4).

**Table 2:** Results of average differences in each concentration group.

Comparison between groups	*Fusobacterium nucleatum* (FN)	*Aggregatibacter actinomycetemcomitans* (AA)	*Porphyromonas gingivalis (PG)*
0.4 and 0.6	0,282	0,625	0,994
0.4 and 0.8	0,003∗	0,041∗	0,733
0.4 and Control (+)	0,000∗	0,966	0,019∗
0.4 and Control (−)	0,000∗	0,000∗	0,000∗
0.6 and 0.8	0,196	0,472	0,921
0.6 and Control (+)	0,000∗	0,276	0,008∗
0.6 and Control (−)	0,000∗	0,000∗	0,000∗
0.8 and Control (+)	0,000∗	0,010∗	0,001∗
0.8 and Control (−)	0,000∗	0,000∗	0,000∗
Control (+) and Control (−)	0,000∗	0,000	0,000∗

∗p value < 0,05.

**Table 3:** MIC value results of the Microdilution Method with TTC Confirmatory test MIC Chitosan Sea Urchins.

Bacteria	256 ppm	128 ppm	64 ppm	32 ppm	16 ppm	8 ppm	4 ppm	2 ppm	1 ppm	0,5 ppm	0,25 ppm	0 ppm
*Fusobacterium nucleatum*	+	+	+	+	+	–	–	–	–	–	–	–
*Aggregatibacter actinomycetemcomitans*	+	+	+	+	–	–	–	–	–	–	–	–
*Porphyromonas gingivalis*	+	+	+	+	–	–	–	–	–	–	–	–

**Table 4:** MBC value results using the scratch method MBC Chitosan Sea Urchins.

Bacteria	256 ppm	128 ppm	64 ppm	32 ppm	16 ppm	8 ppm	4 ppm	2 ppm	1 ppm	0,5 ppm	0,25 ppm	0 ppm
*Fusobacterium nucleatum*	+	+	+	+	+	–	–	–	–	–	–	–
*Aggregatibacter actinomycetemcomitans*	+	+	+	+	+	+	+	–	–	–	–	–
*Porphyromonas gingivalis*	+	+	+	–	–	–	–	–	–	–	–	–

+ = Inhibits microbial growth (does not turn red after being dropped on TTC).

- = Does not inhibit microbial growth (turns red after being dropped on TTC).

### 3.3 SEM-EDX sea urchin's chitosan

**Fig. 6:**
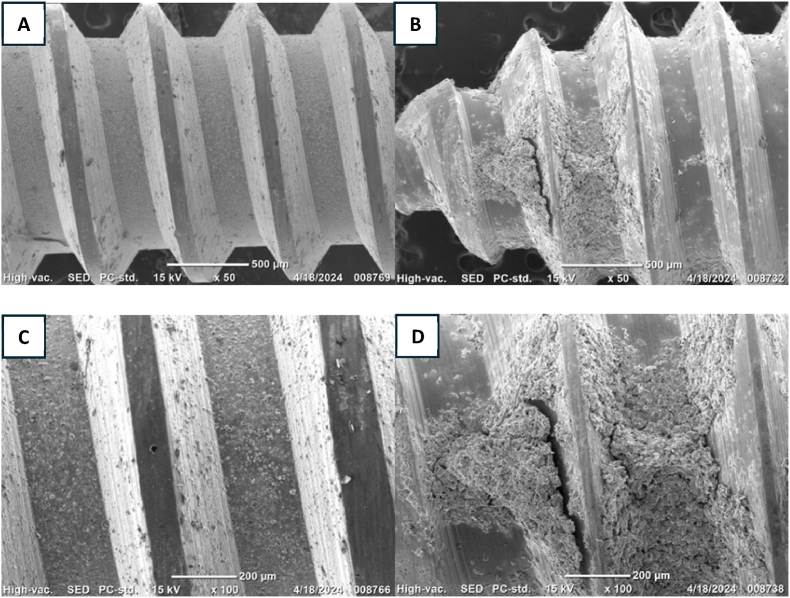
Surface characterization of the miniscrew scanning electron micrographs. (A, B) 500 μm with 50x magnification. (D, E) 200 μm with 100x magnification.

The SEM images of miniscrew coated in chitosan revealed a mesoporous/macroporous surface structure, demonstrating effective adherence to the surfaces of orthodontic miniscrews (see Fig. 6).

## 4 Discussion

Our study thoroughly investigated the use of chitosan to enhance the antimicrobial properties of coated miniscrews. The characterization of prepared chitosan by FTIR spectra validates the presence of chitosan characteristics within the compound extracted from sea urchins.^24^ The EDX analysis spectrum, illustrated in Fig. 2, assesses the Ca/P ratio for the synthesized chitosan-based calcium compound. This analysis confirms the presence of C, N, and O within the chitosan matrix. These findings are further corroborated by SEM imagery in Fig. 3, which reveals agglomerated and porous molecular structures.^31^

The novel chitosan demonstrated promising antimicrobial effects against three bacterial strains: *A. actinomycetemcomitans, F. nucleatum,* and *P. Gingivalis,* which showed the most excellent inhibition zones at a concentration of 0.8 %, decreasing the inhibition zones as the concentration was reduced. The minimum inhibitory concentration (MIC) values varied according to the bacterial strains, with F. nucleatum having an MIC of 16 ppm and *A. actinomycetemcomitans* and *P. gingivalis* having an MIC of 32 ppm each. The minimum bactericidal concentration (MBC) values were 4 ppm for *A. actinomycetemcomitans*, 16 ppm for *F. nucleatum*, and 64 ppm for *P. gingivalis*. These results are supported by previous studies indicating the effectiveness of sea urchin chitosan against various bacterial pathogens^24^^,^^25^

A prior study indicated that various factors influence chitosan's antibacterial efficacy. These include microbial characteristics, such as species and population size; intrinsic properties of chitosan, including molecular weight, concentration, and degree of deacetylation; and physical variables like chitosan solubility in water. Additionally, environmental conditions—such as pH level, temperature, and reaction duration—also play a significant role in its effectiveness.^32^

The antibacterial effectiveness of chitosan against Gram-negative bacteria, specifically *A. actinomycetemcomitans, F. nucleatum,* and *P. gingivalis*, presents both advantages and disadvantages. Previous research indicates that chitosan demonstrates reduced efficacy against Gram-negative bacteria, which may be attributed to their unique structural characteristics. Research suggests a potential greater susceptibility of gram-negative bacteria to chitosan compared to gram-positive bacteria.^33^^,^^34^ However, certain studies have demonstrated that gram-positive bacteria exhibit a higher sensitivity to chitosan.^35^ Unlike Gram-positive bacteria that possess only a thick peptidoglycan layer containing teichoic acid (which is negatively charged), Gram-negative bacteria feature three protective barrier membranes: the hydrophobic outer membrane, the peptidoglycan layer, and the inner cell membrane.^36^ The results of this study are in line with previous research that mentioned chitosan can inhibit the growth of these three strain bacterias.^37^^,^^38^ In this study, the antibacterial efficacy of the chitosan group was found to be less effective than that of the azithromycin group. This observation may be attributed to the fact that the antibacterial mechanism of chitosan is primarily bacteriostatic; it acts by destabilizing cell membranes rather than directly killing bacteria.^39^

The increasing incidence of bacterial resistance, combined with a decline in innovative antibiotic development, has significantly altered the medical community's approach to managing infections.^18^ Historically, strategies centered on completely eradicating bacteria; however, contemporary methods are shifting toward using biomaterials that exhibit a reduced propensity for inducing bacterial resistance. These advanced materials serve to mitigate bacterial virulence through various mechanisms, including the inhibition of toxin production, restriction of nutrient access for bacteria, and enhancement of host immune responses.^40^^,^^41^ Chitosan obtained from sea urchins, which are plentiful in Indonesia, shows considerable promise as an antimicrobial agent specifically targeted at inhibiting biofilm development.

## 5 Conclusion

This study demonstrates that chitosan derived from *Diadema setosum* spines shows significant promise as an antimicrobial coating for orthodontic miniscrews, effectively combating peri-implantitis-associated pathogens. The chitosan coating displayed notable antibacterial activity, with the ability to inhibit key Gram-negative bacteria, including *Aggregatibacter actinomycetemcomitans, Fusobacterium nucleatum,* and *Porphyromonas gingivalis.* The results highlight the potential of chitosan to disrupt bacterial cell membranes and inhibit biofilm formation, which is essential for reducing the risk of infection and enhancing the longevity of miniscrew applications in orthodontics. Given its natural origin and antimicrobial properties, chitosan from sea urchin spines presents a valuable alternative to conventional antibiotics, especially in the context of rising antibiotic resistance.

## 6 Limitations and future directions

While this study highlights the potential of sea urchin-derived chitosan coatings, the lack of in vivo studies limits the understanding of its clinical efficacy, biocompatibility, and long-term safety. Future animal studies, including histopathological analyses, are needed to evaluate its effects on surrounding tissues and its mechanism of action. These findings will be critical in bridging the gap between in vitro research and human trials, paving the way for clinical applications in managing peri-implantitis.

## Ethical clearance

This experiment was performed in accordance with the International Guidelines for the care and use of laboratory animals and plants. Clearance for this study was obtained from Hasanuddin University Dental Hospital, Makassar, Indonesia; Research Ethics Committee number: **018/KEPK-FKG-RSGMP-UH/EA/X2024.**

## Patients declarations

The study is based on in vitro experiments and does not involve human or animal subjects. Our findings suggest significant promise for sustainable biomaterials in infection control, which we believe will interest your readership.

## Sources of funding

This study was funded by Penelitian Fundamental Kolaboratif (Grant No: 00309/UN 4.22/PT.01.03/2024), Hasanuddin University, Makassar, Indonesia.

## Declaration of competing interest

The authors declare that they have no known competing financial interests or personal relationships that could have appeared to influence the work reported in this paper.
